# MemSAM-2.5D: overcoming volumetric discontinuity and boundary ambiguity for 3D liver tumor segmentation

**DOI:** 10.3389/fonc.2026.1850004

**Published:** 2026-07-08

**Authors:** Yinyin Hou, Ningning Chen, Tingting Huo, Weijia Wang

**Affiliations:** 1 Department of Medical Oncology, Shaanxi Provincial People’s Hospital, Xi’an, Shaanxi, China; 2 Department of Medical Oncology, The First Affiliated Hospital of Xi’an Jiaotong University, Xi’an, Shaanxi, China

**Keywords:** hepatocellular carcinoma, liver tumor segmentation, medical image segmentation, memory networks, state space models

## Abstract

**Introduction:**

Accurate segmentation of liver tumors from 3D computed tomography (CT) volumes is essential for the clinical management of hepatocellular carcinoma (HCC), but remains challenging because of extreme lesion-scale variation, volumetric discontinuity across slices, and ambiguous tumor boundaries.

**Methods:**

We propose MemSAM-2.5D, a unified 2.5D segmentation framework built upon the MedSAM foundation model. The framework integrates a Hybrid Mamba-Adapter (HMA) for intra-slice multi-scale representation, a Z-axis State Flow (ZSF) module for continuous inter-slice dependency modeling, and a Confidence-Gated Prototype Memory (CGPM) module for uncertainty-aware boundary refinement.

**Results:**

Extensive evaluations on MSD08, HCC-TACE-Seg, and WAW-TACE demonstrate that MemSAM-2.5D consistently outperforms representative CNN-based, Transformer-based, Mamba-based, and MedSAM-based baselines. The improvements are reflected not only in overlap-based metrics, but also in boundary-sensitive, lesion-level, and continuity-related measures.

**Discussion:**

These results suggest that coordinated modeling of multi-scale lesion variability, z-axis continuity, and boundary ambiguity provides an effective and transferable solution for clinically relevant HCC segmentation in CT volumes.

## 1 Introduction

HCC represents a significant global health burden Lee (1) Pan et al. (2) Han et al. (3). Accurate segmentation of liver tumors from 3D CT volumes plays a pivotal role in clinical diagnosis, treatment planning, and prognostic evaluation.

Despite its clinical importance, manual delineation of liver tumors remains highly time-consuming and subjective. In clinical practice, radiologists routinely encounter three major diagnostic challenges. First, liver tumors exhibit extreme scale variations, ranging from subtle local enhancements to massive lesions Wang et al. (4) Amiri et al. (5). Second, volumetric CT scans inherently possess continuous contextual dependencies along the Z-axis, but independent slice-by-slice assessment often leads to discontinuous predictions and abrupt area fluctuations Reiazi et al. (6). Third, HCC lesions frequently present ambiguous boundaries with infiltrative growth patterns, significantly increasing the risk of false positives during diagnosis Li et al. (7) Napolitano et al. (8).

To alleviate the clinical burden, deep learning algorithms have been widely introduced to assist in tumor segmentation Gul et al. (9) Montalbo et al. (10); Hariharan et al. (11). However, existing computational approaches exhibit several critical limitations. For intra-slice modeling, conventional Convolutional Neural Networks (CNNs) lack global receptive fields Li et al. (12) Xu et al. (13), whereas pure Transformer architectures introduce substantial computational burdens, failing to efficiently balance multi-scale feature extraction Afridi et al. (14) Hamamci et al. (15). For inter-slice modeling, direct 3D networks consume excessive memory, while 2.5D models often struggle to efficiently capture long-range sequential dependencies along the Z-axis Li et al. (16). Furthermore, regarding ambiguous boundaries, most existing models generate deterministic predictions without evaluating localization uncertainty Han et al. (17) Li et al. (18) Yang et al. (19). When ambiguous features are indiscriminately aggregated into temporal modules or memory banks, they severely contaminate the target representation, ultimately yielding unreliable contour delineations.

To bridge these research gaps, we propose MemSAM-2.5D, a unified 2.5D segmentation framework built upon the MedSAM foundation model. Our framework seamlessly integrates three task-oriented enhancements to address the aforementioned clinical pain points. First, we embed a HMA into the frozen image encoder, effectively capturing multi-scale contextual correlations without heavy parameter overhead. Second, we propose a ZSF module to model long-range sequential dependencies, utilizing a one-dimensional state-space model to propagate tumor-aware latent states across adjacent slices. Finally, we introduce a CGPM module. By evaluating the predictive uncertainty of intermediate features, this module constructs a reliable prototype memory bank that explicitly excludes ambiguous boundary regions, ensuring robust boundary refinement.

Extensive experiments on the MSD08 benchmark and two HCC-specific cohorts demonstrate that MemSAM-2.5D significantly outperforms state-of-the-art methods. The main contributions of this work are summarized as follows:

- We propose MemSAM-2.5D, an efficient and unified framework that adapts the foundation model SAM for 3D liver tumor segmentation, effectively addressing multi-scale variation, volumetric discontinuity, and boundary ambiguity.
- We design a HMA that adaptively integrates local convolutional textures and global state-space context, enhancing intra-slice multi-scale feature representation.
- We introduce a ZSF module that effectively maintains volumetric continuity by propagating compact latent states across adjacent slices, avoiding the severe memory consumption of full 3D convolutions.
- We develop a CGPM module that leverages uncertainty estimation to identify reliable target states, preventing memory contamination and effectively refining ambiguous tumor boundaries.
- Comprehensive evaluations demonstrate that MemSAM-2.5D achieves state-of-the-art performance on both general liver tumor benchmarks and challenging HCC-specific datasets.

## 2 Related work

### 2.1 Foundation models and adapters in medical image segmentation

The Segment Anything Model (SAM) Kirillov et al. (20) has demonstrated remarkable zero-shot generalization in natural images, but its direct application to medical images often suffers from significant domain shifts Mazurowski et al. (21). To bridge this gap, MedSAM Ma et al. (22) fine-tuned SAM specifically for diverse medical image datasets. To mitigate the computational burden and overfitting risks of full fine-tuning, parameter-efficient adaptation strategies have been widely adopted. For instance, Medical SAM Adapter Wu et al. (23) integrates lightweight trainable modules into the frozen SAM backbone, efficiently transferring pre-trained semantic knowledge to specific medical tasks.

### 2.2 State space models for volumetric dependencies

Volumetric medical image segmentation requires effective modeling of inter-slice and intra-slice dependencies. Traditional 3D CNNs Çiçek et al. (24) and Vision Transformers [e.g., UNETR Hatamizadeh et al. (25)] incur severe memory consumption and quadratic computational complexity. Recently, State Space Models (SSMs) like Mamba Gu and Dao (26) have emerged as a powerful alternative for long-range sequence modeling with linear complexity. Researchers have adapted Mamba for medical vision, developing architectures such as VM-UNet Ruan et al. (27) and SegMamba Xing et al. (28), which apply spatial Mamba blocks to capture global volumetric correlations. However, efficiently balancing local texture preservation and global state propagation in a 2.5D sequential framework remains an open challenge.

### 2.3 Uncertainty estimation and memory networks

Medical lesions frequently exhibit ambiguous boundaries, making deterministic segmentation predictions unreliable. Uncertainty estimation Kendall and Gal (29) is a crucial mechanism to quantify predictive ambiguity and evaluate model trustworthiness Danelljan et al. (30). Concurrently, memory networks Yang and Chan (31) are utilized to dynamically store and retrieve historical target representations to maintain stability over time. Recent tracking and segmentation methodologies have begun integrating these paradigms Yao et al. (32). By using uncertainty to gauge localization confidence, models can construct prototype memory banks that selectively store only highly reliable features, explicitly preventing ambiguous boundary representations from contaminating the target state.

## 3 Method

### 3.1 Overall framework

The objective of MemSAM-2.5D. HCC segmentation in CT volumes presents three primary challenges. First, lesions exhibit extreme multi-scale variations. Second, independent 2D slice predictions cause abrupt inconsistency across adjacent slices. Third, ambiguous tumor boundaries frequently lead to false positives. To address these issues, we propose MemSAM-2.5D. This unified 2.5D segmentation framework is built upon the well-established MedSAM-B backbone.

**Figure 1:**
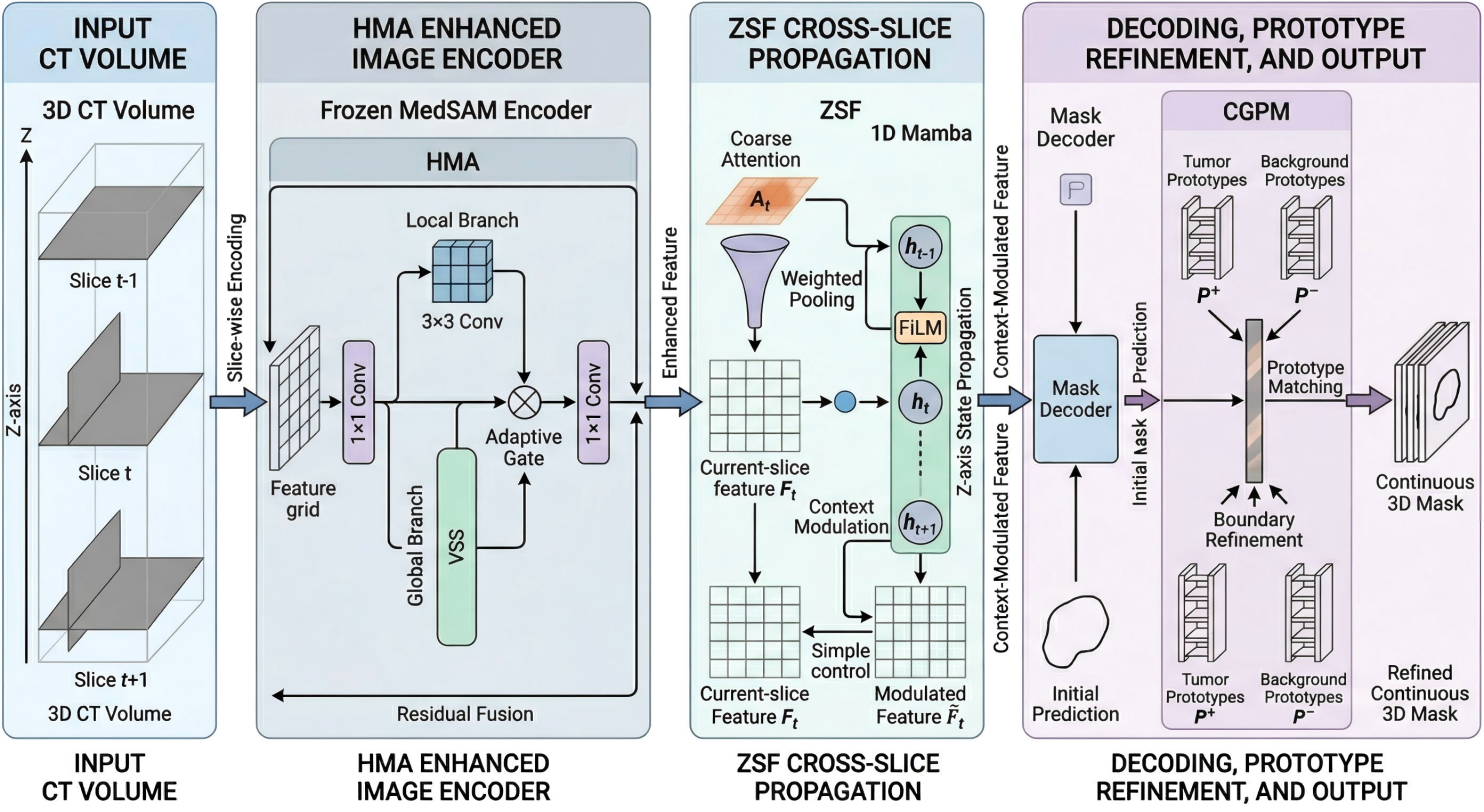
Overview of the proposed MemSAM-2.5D framework for hepatocellular carcinoma (HCC) segmentation in computed tomography (CT) volumes. The input 3D CT volume is processed slice by slice along the z-axis. The HMA-enhanced image encoder strengthens intra-slice representation by combining local detail extraction and global contextual modeling. The ZSF module captures cross-slice continuity by propagating tumor-aware latent states and modulating the current-slice features. The mask decoder then generates an initial prediction, which is further refined by the class-guided prototype memory (CGPM) using reliable tumor and background prototypes. The final output is a continuous and refined 3D segmentation mask. HMA, hybrid multi-scale adapter; ZSF, z-axis state fusion; CGPM, class-guided prototype memory.

The inputs, outputs, and details of MemSAM-2.5D. As illustrated in **Figure 1**, the proposed framework takes a 3D CT volume $V\in {\mathbb{R}}^{D\times H\times W}$ as input. The network processes this volume slice by slice along the depth dimension *D*. MemSAM-2.5D preserves the original architecture of MedSAM, which includes an image encoder, a prompt encoder, and a mask decoder. We introduce three task-oriented enhancements into this backbone. First, a HMA enhances multi-scale contextual correlations within each independent slice. Second, a ZSF module propagates tumor-aware latent states across adjacent slices to maintain volumetric continuity. Finally, a CGPM module refines ambiguous boundaries using high-confidence class prototypes. Consequently, MemSAM-2.5D outputs a continuous and refined 3D segmentation mask sequence. This pipeline integrates intra-slice modeling, inter-slice state propagation, and boundary refinement into a single promptable workflow.

### 3.2 HMA

The objective of HMA. HCC lesions exhibit marked scale variations, ranging from subtle local enhancements to massive tumors spanning the liver. Conventional convolutional adapters possess a limited receptive field. Global Transformer adapters introduce substantial computational burdens. Therefore, we design a HMA to achieve an optimal balance between global dependency modeling and computational efficiency.

The inputs, outputs, and details of HMA. We embed the HMA into the frozen MedSAM image encoder. The input to this module is a feature map ${\bar{X}}_{t}^{l}\in {\mathbb{R}}^{H_{l}\times W_{l}\times C_{l}}$ extracted from the *l*-th Vision Transformer (ViT) block. For the MedSAM-B backbone, the channel dimension *C_l_*is 768. The module first applies a 1 × 1 convolution to project this input into a lower-dimensional bottleneck space. This projection reduces the channel dimension from 768 to 64, yielding the bottleneck feature $U_{t}^{l}$. The HMA then processes this bottleneck feature through two parallel branches. The local branch employs depthwise separable convolutions with a kernel size of 3 × 3 to preserve fine lesion textures and edges. Concurrently, the global branch leverages a Visual State-Space (VSS) module to capture long-range contextual correlations across the entire slice. To adaptively integrate local details and global context, the network computes a spatial gate $g_{t}^{l}$. This gate is generated by applying Global Average Pooling (GAP) and a Multi-Layer Perceptron (MLP) to the concatenated branch features. The gate is computed as shown in Equation 1.

(1) $$g_{t}^{l}=\sigma \left(\text{MLP}\left(\text{GAP}\left(\left[U_{t,\text{loc}}^{l};U_{t,\text{glo}}^{l}\right]\right)\right)\right).$$

The gate $g_{t}^{l}$ then controls the element-wise fusion of the two branches to produce the output feature ${\hat{U}}_{t}^{l}$. The two branches are fused according to Equation 2.

(2) $${\hat{U}}_{t}^{l}=g_{t}^{l}⊙U_{t,\text{glo}}^{l}+\left(1-g_{t}^{l}\right)⊙U_{t,\text{loc}}^{l}.$$

Finally, the module projects the fused feature ${\hat{U}}_{t}^{l}$ back to the original 768 dimensions using a 1 × 1 convolution. The network adds this expanded feature to the original encoder feature via a residual connection. This final output replaces the original feature map for the subsequent ViT blocks.

### 3.3 ZSF

The objective of ZSF. Processing 2D slices independently ignores the continuous volumetric nature of HCC. This independence leads to fragmented predictions and abrupt area fluctuations across adjacent slices. Full 3D networks mitigate this issue but incur severe memory consumption. To maintain volumetric continuity efficiently, we propose the ZSF module. This module models the CT volume along the Z-axis as a continuous sequence of compressed latent states.

The inputs, outputs, and details of ZSF. The input to the ZSF module is the HMA-enhanced encoder feature $F_{t}\in {\mathbb{R}}^{C\times h\times w}$ from the current slice. Here, *C* = 256, and *h* = *w* = 64. The module first applies a 1×1 convolution followed by a Sigmoid function to generate a coarse segmentation probability map *A_t_*. Utilizing *A_t_* as a spatial attention guide, the network performs weighted global average pooling on *F_t_*. This pooling operation distills a tumor-aware summaryvector $z_{t}\in {\mathbb{R}}^{256}$. This vector encapsulates the lesion-specific representation of the current slice *t*. Next, the module propagates this latent state along the Z-axis using a one-dimensional Mamba block: $h_{t}={\text{Mamba}}_{z}\left(z_{t},h_{t-1}\right)$. The hidden state *h_t_*_−1_ encapsulates the tumor context compressed from all preceding slices. The propagated state *h_t_*_−1_ then modulates the current slice feature *F_t_* before the decoding stage. We employ a Feature-wise Linear Modulation (FiLM) transformation for this process. The module computes the affine parameters *γ_t_* and *β_t_* using a two-layer MLP, and applies them to generate the final output feature ${\tilde{F}}_{t}$. The affine parameters are computed using Equation 3 and The context-modulated feature is obtained using Equation 4.

(3) $$\left[\gamma_{t},\beta_{t}\right]=\text{MLP}\left(h_{t-1}\right),$$

(4) $${\tilde{F}}_{t}=\left(1+\gamma_{t}\right)⊙F_{t}+\beta_{t}.$$

By propagating only a 256-dimensional compact latent state, the ZSF module yields the context-modulated feature ${\tilde{F}}_{t}$. The network passes this output directly to the mask decoder.

### 3.4 CGPM

The objective of CGPM. HCC lesions frequently present ambiguous boundaries and infiltrative growth patterns. Uncertain boundary features can contaminate the target representation if the network indiscriminately writes all predicted features into a memory bank. This contamination yields severe false positives in subsequent slices. To identify reliable target states and refine boundary predictions, we introduce the CGPM module.

The inputs, outputs, and details of CGPM. The CGPM maintains two compact sets of high-confidence class prototypes: a tumor-core set *P*^+^ and a safe-background set *P*^−^. Each class memory stores *K* prototype vectors of dimension 32, where *K* = 8 is used as the default setting according to the sensitivity analysis. The inputs to this module are the intermediate decoder feature $G_{t}\in {\mathbb{R}}^{32\times h\times w}$ and the raw logit prediction $\ell_{t}$. The module evaluates the predictive uncertainty of *G_t_* using information entropy *H_i_* calculated from the logit probabilities at each spatial position *i*. To construct a reliable memory bank, the CGPM applies a strict confidence-gating mechanism. The network only permits features with high prediction confidence (*p_i_* > 0.9) and low uncertainty (*H_i_* < 0.2) to update the prototypes. This update uses an exponential moving average with a momentum of 0.99. During the inference phase, the module reads from this reliable memory to correct ambiguous regions. For each spatial position *i*, the network computes its maximum cosine similarity to both the tumor prototypes ( $s_{i}^{+}$) and the background prototypes ( $s_{i}^{-}$). Driven by an entropy-based read gate $u_{i}=H_{i}/\text{log }2$, the module applies a posterior correction to the original logit $\ell_{i}$. The posterior correction is formulated in Equation 5.

(5) $${\ell_{i}}^{\prime }=\ell_{i}+\lambda_{m}\;u_{i}\;\left(s_{i}^{+}-s_{i}^{-}\right).$$

We set the memory impact weight λ*_m_* to 0.5. The final output of the CGPM is the refined logit ${\ell_{i}}^{\prime }$. This output forces ambiguous boundary pixels to align with the most similar reliable historical prototypes.

## 4 Experimental results

### 4.1 Datasets, compared methods, and implementation details

#### 4.1.1 Datasets

To comprehensively evaluate the proposed method in both general liver tumor segmentation and HCCspecific segmentation scenarios, we conducted experiments on one public liver tumor benchmark and two HCC-focused datasets. Specifically, MSD08 (Medical Segmentation Decathlon Task08) was adopted for the general liver tumor setting, while HCC-TACE-Seg and WAW-TACE were used for the HCC-specific setting. In addition, the LiTS 2017 dataset was incorporated as an auxiliary training set during model development to improve the diversity of liver tumor appearances observed during training.

For the general liver tumor benchmark, we used the MSD08 dataset, which contains 303 CT volumes. According to prior studies, 161 volumes are provided with pixel-wise Couinaud segment annotations and are commonly split into 113, 16, and 32 cases for training, validation, and testing, respectively, whereas the remaining 142 volumes are often used as an additional test subset. Because MSD08 has been widely adopted in recent liver tumor SAM adaptation studies, it is well suited for direct comparison with methods such as CouinaudSAM.

In addition to MSD08, we also used the LiTS 2017 dataset as an auxiliary training source. LiTS provides contrast-enhanced abdominal CT scans with liver and liver tumor annotations and has been widely used in liver tumor segmentation research. In this study, LiTS was used only to enrich the training distribution and was not treated as a separate evaluation benchmark in the main tables.

For the HCC-specific setting, HCC-TACE-Seg was used as the primary internal evaluation cohort. This dataset contains CT scans from 105 confirmed HCC patients collected at MD Anderson Cancer Center. It includes multiphase contrast-enhanced imaging together with liver and tumor annotations. Recent public benchmarks typically use only the portal-venous phase and adopt an 85/10/10 split for training, validation, and testing. Notably, the cases in this dataset are explicitly restricted to solitary tumors, making it particularly suitable for evaluating boundary delineation and lesion contour stability.

To further assess external generalization, we adopted WAW-TACE as an independent external test cohort. The original cohort includes multiphase CT scans from 233 treatment-naive HCC patients. Recent reports indicate that only 74 portal-venous phase scans provide annotations directly usable for tumor segmentation evaluation. In addition, the tumor pixel ratio in WAW-TACE is only approximately 0.16%, which is substantially lower than the 1.22% reported for HCC-TACE-Seg. Therefore, this dataset is more challenging and better reflects model robustness under severe class imbalance and cross-center domain shifts. In our experiments, all 74 portal-venous phase scans were reserved exclusively for external testing and were not used for training or hyperparameter selection.

In summary, MSD08 was used for evaluating general liver tumor segmentation, HCC-TACE-Seg was used for model development and internal testing, and WAW-TACE was used only for external generalization assessment. LiTS 2017 was additionally used as an auxiliary training dataset and was not included as an independent test benchmark in the main comparison tables. Since the portal-venous phase has been regarded in recent liver tumor segmentation benchmarks as the most stable and consistently available phase for distinguishing liver parenchyma from tumor tissue, all HCC-specific experiments were conducted using the portal-venous phase.

#### 4.1.2 Compared methods

To ensure a sufficiently comprehensive comparison, we selected representative baselines from four categories, namely CNN-based models, Transformer-based models, Mamba-based models, and foundationmodel adaptation methods.

Specifically, nnU-Net was used as a strong CNN baseline, SwinUNETR was included as a representative 3D Transformer baseline, and U-Mamba was adopted as a recent CNN–SSM hybrid baseline. These three model families have also been identified in recent HCC segmentation benchmarks as representative solutions for liver tumor segmentation. In addition, because our method is built upon MedSAM, MedSAM-B was included as the direct foundation-model baseline. For MSD08, we further included CouinaudSAM, as it is among the most relevant publicly reported SAM-based comparison methods for liver tumor segmentation and provides relatively complete quantitative results on MSD08.

MedSAM was pretrained on 1,570,263 image–mask pairs covering 10 imaging modalities and more than 30 cancer types. It therefore serves as a reasonable reference for evaluating how well a general medical foundation model can be transferred to HCC segmentation. In the HCC-specific experiments, we also included a MedSAM-B + CNN Adapter reproduction baseline to more directly examine whether a lightweight adapter alone is sufficient, and to provide a more architecture-aligned comparison with the proposed MemSAM-2.5D.

It should be noted that the CouinaudSAM results on MSD08 were taken directly from the original protocol in order to avoid additional implementation bias caused by differences in Couinaud prompt generation. By contrast, the MedSAM-series results in the HCC-specific experiments were reimplemented under a unified automatic liver-box prompt setting.

#### 4.1.3 Implementation details

Our method uses MedSAM-B as the unified backbone. For all MedSAM-based methods, a frozen liver localizer was first used to generate a coarse liver mask, from which a liver ROI bounding box was extracted. This box was used both for input cropping and as the automatic box prompt for the prompt encoder. Each sample was constructed as a 2.5D input using three adjacent slices, i.e., [*x_t_*_−1_,*x_t_*,*x*_*t* + 1_]. All CT volumes were first cropped to the liver region and then resized to 512×512. For MSD08, following the preprocessing convention of CouinaudSAM, Hounsfield units were clipped to [−200,250] and then normalized. The same intensity window was adopted in the HCC-specific experiments to ensure consistency between training and testing.

During training, the MedSAM image encoder was frozen, and only the HMA, ZSF, CGPM, mask decoder, and the learnable tumor token were updated. HMA modules were inserted after the 4th, 8th, and 12th ViT blocks. The default Z-axis chunk length was set to 8. The numbers of tumor and background prototypes were both set to 8. The confidence threshold *τ_p_*was set to 0.9, and the entropy threshold *τ_h_*was set to 0.2. We used the AdamW optimizer with an initial learning rate of 1 × 10^−4^ and a weight decay of 1 × 10^−4^. The total number of training epochs was 100, with the first 10 epochs used for warm-up.

Following the method definition, the training objective consisted of a segmentation loss and an auxiliary loss for the coarse head. The segmentation loss combined Dice loss and BCE loss, while the auxiliary Dice loss was applied to the coarse prediction used by ZSF. All experiments were implemented in PyTorch and MONAI. Unless otherwise specified, all in-house trained models were run three times with different random seeds, and the average performance is reported.

For the 3D baselines, nnU-Net was trained using the 3D full-resolution configuration, SwinUNETR followed the publicly recommended volumetric setting, and U-Mamba used its bottleneck variant. These settings are consistent with recent liver tumor and HCC benchmark implementations.

#### 4.1.4 Evaluation metrics

On MSD08, Dice Similarity Coefficient (DSC) and Normalized Surface Dice (NSD) were used as the primary evaluation metrics for direct comparability with CouinaudSAM and related work.

For the HCC-specific experiments, we additionally reported Dice, NSD, the 95th percentile Hausdorff Distance (HD95), and lesion-wise F1 score, considering that HCC lesions are often characterized by severe class imbalance, small lesions that are easy to miss, and ambiguous tumor boundaries. Lesion-wise F1 was computed based on 3D connected components, where a predicted lesion was counted as correctly detected if it had any non-zero voxel overlap with a ground-truth lesion. In the ablation study, we further included FP/case and Slice Area Consistency Error (SACE) to quantify the number of false positives and the inter-slice consistency of predicted lesion area changes, respectively.

#### 4.1.5 Statistical analysis, clinical utility evaluation, and inference benchmark

To assess whether the observed performance gains were statistically meaningful at the case level, we performed paired non-parametric Wilcoxon signed-rank tests between MemSAM-2.5D and the two strongest baseline methods on each HCC-specific cohort. Bonferroni correction was applied within each dataset over the two pre-specified baseline comparisons. Statistical tests were conducted on case-wise Dice, NSD, HD95, and lesion-wise F1 values, and the corrected exact p-values are reported.

To further evaluate downstream clinical utility, we measured tumor-volume estimation consistency. For each test case, the predicted tumor volume was computed as the number of predicted tumor voxels multiplied by the physical voxel spacing. We then compared algorithm-derived tumor volumes with radiologist-annotated reference tumor volumes using Pearson correlation coefficient (*R*) and mean absolute percentage error (MAPE).

To further quantify robustness to liver-ROI localization errors, we simulated controlled perturbations of the automatic liver ROI box without retraining. For box-shift perturbation, the ROI center was shifted by 5% or 10% of the box side length along the in-plane directions while keeping the box size unchanged. For tight-cropping perturbation, the box width and height were reduced by 5% or 10% in total while keeping the box center fixed. Results were averaged over perturbation directions and reported as Dice degradation relative to the unperturbed automatic ROI setting.

Finally, to evaluate deployment feasibility, we benchmarked inference latency and peak GPU memory usage for nnU-Net, U-Mamba, and MemSAM-2.5D under the same liver-ROI preprocessing setting. For MemSAM-2.5D, latency was measured over the sequential liver-ROI slice sequence with Z-axis state propagation enabled. We report total inference time per liver-ROI volume, effective latency per processed ROI slice, and inference peak GPU memory.

### 4.2 Main results

#### 4.2.1 Results on the general liver tumor benchmark (MSD08)

As shown in **Table 1**, three observations can be made. First, directly transferring MedSAM-B to liver tumor segmentation yields reasonably competitive results, but it still falls noticeably behind CouinaudSAM on MSD08. This suggests that generic medical foundation-model pretraining alone is insufficient to fully address the complexity of liver tumors in terms of scale variation, spatial location, and boundary ambiguity.

**Table 1:** Quantitative comparison on MSD08.

Method	MSD08-161 DSC (%) ↑	MSD08-161 NSD (%) ↑	MSD08-142 DSC (%) ↑	MSD08-142 NSD (%) ↑
UNet++	68.64	56.23	64.50	71.19
nnU-Net	68.19	56.03	64.78	71.24
DAEFormer	70.49	58.06	66.26	73.83
CouinaudSAM-B	72.98	61.41	70.82	76.81
CouinaudSAM-H	73.52	63.02	72.50	79.40
MedSAM-B	70.53	58.61	67.88	74.37
MemSAM-2.5D (Ours)	**74.81**	**64.24**	**73.29**	**80.06**

Bold values indicate the best performance among all compared methods or settings. ↑ indicates that higher values are better.

Second, the proposed method consistently outperforms both MedSAM-B and CouinaudSAM on the two MSD08 subsets. Compared with CouinaudSAM-H, MemSAM-2.5D improves DSC by 1.29 points on MSD08–161 and by 0.79 points on MSD08-142, while also yielding clear gains in NSD. This pattern indicates that the improvement is not merely due to coarse region expansion, but is also accompanied by better boundary alignment.

Third, although CouinaudSAM explicitly uses anatomical prompting, the proposed method still achieves stronger overall performance without relying on such prompts. This result suggests that the combination of enhanced intra-slice long-range modeling, inter-slice state propagation, and high-confidence boundary correction can compensate for the absence of explicit anatomical guidance to a considerable extent.

#### 4.2.2 Results on HCC-specific internal and external cohorts

**Table 2** shows that the proposed method achieves the best performance on both the internal and external test cohorts, and the improvements are consistent across all major evaluation dimensions. The gains are not limited to region-overlap metrics such as Dice, but are also reflected in the boundary-sensitive metric HD95 and the lesion-level detection metric.

**Table 2:** Quantitative comparison on HCC-TACE-Seg and WAW-TACE.

Method	HCC-TACE				WAW-TACE			
	Dice(%) ↑	NSD(%) ↑	HD95(mm) ↓	Lesion-F1(%) ↑	Dice(%) ↑	NSD(%) ↑	HD95(mm) ↓	Lesion-F1(%) ↑
nnU-Net	76.4	66.9	11.2	75.4	74.6	63.8	12.9	72.9
SwinUNETR	72.8	61.5	15.4	70.2	69.8	58.1	17.6	67.0
U-Mamba	75.9	66.0	11.9	74.8	73.8	63.1	13.4	72.2
MedSAM-B	72.1	60.8	14.9	70.4	70.4	58.9	16.7	68.5
MedSAM-B + CNN Adapter	76.8	67.0	11.0	75.8	75.0	64.1	12.2	73.3
MemSAM-2.5D (Ours)	**79.0**	**69.5**	**9.6**	**78.4**	**77.1**	**66.2**	**10.8**	**75.4**

Bold values indicate the best performance among all compared methods or settings. ↑ and ↓ indicate that higher and lower values are better, respectively.

Compared with the strongest lightweight MedSAM reproduction baseline, MemSAM-2.5D improves Dice by 2.2 points on HCC-TACE-Seg, reduces HD95 by 1.4 mm, and increases lesion-wise F1 by 2.6 points. On the external WAW-TACE cohort, it maintains a Dice advantage of 2.1 points and also improves the boundary and lesion-level metrics. Such consistent gains suggest that the proposed method does not simply adapt the mask decoder to one specific data distribution, but instead provides more transferable benefits at the levels of feature modeling and discriminative correction.

A comparison across baseline families further reveals that SwinUNETR performs noticeably worse than nnU-Net and U-Mamba on both HCC-specific datasets. This observation is consistent with the general tendency that, in liver tumor and HCC scenarios with limited training data, pure volumetric Transformer models are often less stable than adaptive CNNs or CNN–SSM hybrids. Meanwhile, U-Mamba performs similarly to nnU-Net, indicating that long-range dependency modeling based on state-space modules is indeed valuable. However, simply incorporating Mamba into a U-Net architecture does not automatically resolve prompt bias, slice continuity, or uncertainty contamination at ambiguous boundaries. By introducing HMA, ZSF, and CGPM on top of MedSAM, our method continues to improve the more sensitive lesion-wise F1 and HD95 metrics.

It is also worth noting that WAW-TACE has a substantially lower tumor pixel ratio than HCC-TACE-Seg. Therefore, some performance drop on the external dataset is expected. Nevertheless, the proposed method preserves a relatively stable margin under this more severe class imbalance, indicating stronger robustness to both cross-center variation and extreme foreground sparsity. This property is particularly important for HCC segmentation, since in real clinical practice, small-volume lesions and lesions with poorly defined boundaries are much more likely to cause automated segmentation failure than large, well-defined tumors.

Statistical significance of the HCC-specific results. As shown in **Table 3**, the improvements of MemSAM-2.5D over the strongest baselines remained statistically significant after Bonferroni correction. On HCC-TACE-Seg, MemSAM-2.5D significantly outperformed MedSAM-B + CNN Adapter and nnU-Net across Dice, NSD, HD95, and lesion-wise F1. The statistical evidence was even stronger on the external WAW-TACE cohort, where all corrected p-values remained below 0.05. These results indicate that the observed gains are not merely due to random seed variation or a small number of favorable cases, but are supported by paired case-wise comparisons.

**Table 3:** Statistical significance testing on HCC-specific cohorts.

Dataset	Comparison	Dice	NSD	HD95	Lesion-F1
HCC-TACE-Seg	MemSAM-2.5D vs. MedSAM-B + CNN Adapter	0.018	0.022	0.031	0.026
HCC-TACE-Seg	MemSAM-2.5D vs. nnU-Net	0.012	0.019	0.028	0.021
WAW-TACE	MemSAM-2.5D vs. MedSAM-B + CNN Adapter	0.006	0.009	0.014	0.018
WAW-TACE	MemSAM-2.5D vs. nnU-Net	0.004	0.007	0.011	0.015

Reported values are Bonferroni-adjusted exact p-values. Bonferroni correction was applied within each dataset over the two planned baseline comparisons.

#### 4.2.3 Downstream clinical utility: tumor-volume estimation

Beyond voxel-wise segmentation accuracy, we further evaluated whether the proposed method improved tumor-volume estimation, which is directly relevant to tumor-burden assessment and longitudinal treatmentresponse monitoring. As shown in **Table 4**, all strong baselines achieved high Pearson correlation with the radiologist-annotated reference tumor volume, but MemSAM-2.5D achieved the highest correlation on both HCC-TACE-Seg and WAW-TACE. Because the voxel-wise reference masks were derived from radiologist annotations, the reference volume used in this analysis represents radiologist-annotated tumor volume. Therefore, this experiment evaluates the agreement between automated segmentation-derived tumor burden and radiologist-defined tumor burden. More importantly, MemSAM-2.5D substantially reduced volumeestimation error, decreasing MAPE from 15.8% to 10.9% on HCC-TACE-Seg compared with MedSAM-B + CNN Adapter, and from 18.7% to 13.1% on WAW-TACE. This suggests that the proposed model not only improves segmentation overlap, but also produces more clinically reliable volumetric tumor-burden estimates.

**Table 4:** Agreement between automated and radiologist-annotated tumor-volume estimation on HCCspecific cohorts.

Method	HCC-TACE-Seg Pearson (*R*) ↑	HCC-TACE-Seg MAPE (%) ↓	WAW-TACE Pearson (*R*) ↑	WAW-TACE MAPE (%) ↓
nnU-Net	0.936	17.2	0.921	19.5
U-Mamba	0.931	17.9	0.915	20.2
MedSAM-B + CNN Adapter	0.944	15.8	0.928	18.7
MemSAM-2.5D	**0.969**	**10.9**	**0.957**	**13.1**

Bold values indicate the best performance among all compared methods or settings. ↑ and ↓ indicate that higher and lower values are better, respectively.

#### 4.2.4 Size-stratified lesion analysis

**Table 5** further demonstrates that the advantage of the proposed method is not restricted to a single lesion scale. Instead, it improves both small-lesion recall and large-lesion contour recovery. Compared with MedSAM-B + CNN Adapter, our method improves small-lesion recall by 4.7 points and large-lesion Dice by 2.1 points. This observation is highly consistent with the design motivation of HMA: the local branch preserves fine-grained responses for tiny lesions, whereas the global Mamba branch complements the modeling of large lesion shape and context. In other words, the value of the Hybrid Mamba-Adapter lies not merely in replacing one adapter with another, but in effectively alleviating the scale conflict that is typical in HCC segmentation.

**Table 5:** Size-stratified lesion analysis on HCC-TACE-Seg.

Method	Small Lesion Recall	Medium Lesion Recall	Large Lesion Dice
	(*<*10 mm) ↑	(10–30 mm) ↑	(*>*30 mm) ↑
nnU-Net	61.5	78.3	82.0
U-Mamba	63.2	77.6	81.5
MedSAM-B	57.4	74.9	80.1
MedSAM-B + CNN Adapter	64.4	79.0	82.6
MemSAM-2.5D (Ours)	**69.1**	**82.6**	**84.7**

Bold values indicate the best performance among all compared methods or settings. ↑ indicates that higher values are better.

### 4.3 Ablation study

#### 4.3.1 Component-wise ablation

**Table 6** clearly reflects the distinct functional roles of the three proposed components. First, adding HMA alone produces the largest gain in Dice, indicating that enhanced intra-slice representation is the foundation of the entire framework. It also yields a clear improvement in lesion-wise F1, suggesting that multi-scale modeling directly benefits lesion detection.

**Table 6:** Component-wise ablation on HCC-TACE-Seg.

Variant	HMA	ZSF	CGPM	Dice (%) ↑	HD95 (mm) ↓	Lesion-F1 (%) ↑	FP/case ↓	SACE ↓
MedSAM-B baseline				72.1	14.9	70.4	1.47	0.214
+ HMA	✓			75.8	12.3	74.3	1.19	0.203
+ HMA + ZSF	✓	✓		77.6	10.6	76.6	1.06	0.158
+ HMA + CGPM	✓		✓	77.1	10.9	75.9	0.86	0.196
Full model	✓	✓	✓	**79.0**	**9.6**	**78.4**	**0.74**	**0.149**

Bold values indicate the best performance among all compared methods or settings. ↑ and ↓ indicate that higher and lower values are better, respectively.

Second, when ZSF is added, SACE decreases substantially from 0.203 to 0.158, and this reduction is proportionally more pronounced than the Dice improvement. This result indicates that the main benefit of ZSF lies in improving inter-slice continuity rather than simply increasing regional overlap.

Third, when CGPM is introduced, FP/case decreases from 1.19 to 0.86, whereas SACE improves only modestly. This suggests that CGPM mainly works by suppressing spurious responses near ambiguous boundaries and vessel-adjacent regions, rather than directly changing inter-slice dynamics.

When all three modules are combined, all metrics reach their best values. This non-redundant additive behavior indicates that the proposed framework is not composed of overlapping design choices, but instead addresses segmentation errors from three complementary levels: intra-slice encoding, inter-slice propagation, and posterior correction.

#### 4.3.2 Effect of spatial guidance in ZSF

**Table 7** evaluates whether the spatial guide used for ZSF state extraction is necessary. The comparison between simple GAP and *A_t_*-guided pooling is particularly important because both variants retain the same 1D Mamba propagation. Therefore, the performance gap isolates the effect of spatially tumor-aware state extraction rather than the effect of state-space propagation itself. Coarse-map guidance produced the best practical performance across Dice, NSD, HD95, FP/case, and SACE, whereas ground-truth mask guidance served only as an upper-bound analysis and was not used during actual inference.

**Table 7:** Effect of spatial guidance strategies in ZSF on HCC-TACE-Seg.

ZSF pooling strategy	Tumor-aware spatial guide	Dice (%) ↑	NSD (%) ↑	HD95 (mm) ↓	FP/case ↓	SACE ↓
No ZSF	–	77.1	67.7	10.9	0.86	0.196
Simple Global Average Pooling	None	78.0	68.3	10.3	0.82	0.171
Learned Query Pooling	Learned query vector	78.4	68.7	10.0	0.79	0.164
Coarse Map Guidance (*A_t_*)	Predicted coarse map	**79.0**	**69.5**	**9.6**	**0.74**	**0.149**
Ground-Truth Mask Guidance	Ground-truth mask; upper bound only	79.5	70.0	9.3	0.72	0.144

Bold values indicate the best performance among all compared methods or settings. ↑ and ↓ indicate that higher and lower values are better, respectively.

#### 4.3.3 Effect of adapter design

**Table 8** shows that the plain CNN Adapter uses the fewest parameters but is insufficient for modeling large-scale contextual information. The Transformer Adapter further improves performance, but at the cost of substantially higher GPU memory consumption. The Pure Mamba Adapter achieves a favorable balance between efficiency and effectiveness, yet still underperforms the proposed hybrid design in local boundary preservation.

**Table 8:** Comparison of different adapter designs on HCC-TACE-Seg.

Adapter type	Trainable params (M) ↓	Dice (%) ↑	Small lesion recall (%) ↑	HD95 (mm) ↓	Training peak GPU memory (GB) ↓
CNN Adapter	**5.1**	76.8	64.4	11.0	**10.7**
Transformer Adapter	7.8	77.3	65.0	10.6	13.6
Pure Mamba Adapter	5.7	77.5	66.2	10.5	11.2
Hybrid Mamba-Adapter (Ours)	6.2	**79.0**	**69.1**	**9.6**	11.9

Bold values indicate the best performance among all compared methods or settings. ↑ and ↓ indicate that higher and lower values are better, respectively.

By contrast, the Hybrid Mamba-Adapter introduces only a limited additional memory overhead while achieving the best Dice and small-lesion recall. This finding confirms that the local convolutional branch and the global state-space branch are indeed complementary, and that such complementarity is particularly well suited to the multi-scale challenges of HCC segmentation.

#### 4.3.4 Branch-level analysis of HMA

As shown in **Table 9**, the local-only branch achieved higher small-lesion recall than the global-only branch, indicating that depthwise convolution better preserves subtle local texture and edge evidence for small HCC lesions. In contrast, the global-only branch achieved higher overall Dice and lower HD95, suggesting that VSS-based long-range modeling better captures global lesion extent and contour regularity. The hybrid HMA achieved the best results across all metrics, supporting the complementarity between local texture preservation and global contextual modeling.

**Table 9:** Branch-level analysis of HMA on HCC-TACE-Seg.

HMA variant	Local DW-conv branch	Global VSS branch	Adaptive fusion gate	Dice (%)↑	Small lesion recall<10 mm (%)↑	Medium lesion recall 10–30 mm (%)↑	Large lesion dice >30 mm (%)↑	HD95 (mm)↓	SACE↓
Only Local Branch	✓	–	–	77.7	68.4	81.2	83.1	10.3	0.156
Only Global Branch	–	✓	–	78.2	66.8	81.9	84.2	9.9	0.152
Hybrid HMA	✓	✓	✓	**79.0**	**69.1**	**82.6**	**84.7**	**9.6**	**0.149**

Bold values indicate the best performance among all compared methods or settings. ↑ indicates that higher values are better, and ↓ indicates that lower values are better.

#### 4.3.5 Hyperparameter sensitivity

As shown in **Table 10**, the model is generally stable with respect to chunk length and prototype number. Specifically, an overly short chunk length *T_c_*is insufficient for capturing enough cross-slice context, whereas an overly long chunk length brings only limited gains while increasing computational cost. Therefore, *T_c_*= 8 achieves the best overall trade-off between efficiency and performance.

**Table 10:** Sensitivity analysis of chunk length and prototype number on HCC-TACE-Seg.

Parameter	Setting	Dice (%) ↑	HD95 (mm) ↓	FP/case ↓
Z-axis chunk length (Tc)	4	78.1	10.3	0.82
	8	**79.0**	9.6	**0.74**
	12	78.8	**9.5**	0.76
Prototype number (K)	4	78.5	9.9	0.79
	8	**79.0**	9.6	**0.74**
	16	78.9	**9.4**	0.77

Bold values indicate the best performance among all compared methods or settings. ↑ and ↓ indicate that higher and lower values are better, respectively.

A similar trend is observed for the prototype number *K*. Too few prototypes weaken the representational capacity of the prototype memory, whereas too many prototypes make the distribution overly sparse and lead to diminishing returns. Although a larger prototype set can slightly benefit one boundary-oriented metric, the intermediate setting provides the most balanced overall performance.

**Table 11** further analyzes the entropy threshold used for CGPM memory writing. A very strict entropy threshold admits too few features into the memory bank, whereas a permissive threshold allows unstable boundary features to update the prototypes. The intermediate threshold *τ_h_*= 0.20 yielded the best Dice, lowest FP/case, and lowest SACE, suggesting the best trade-off between prototype reliability and prototype diversity.

**Table 11:** Fine-grained sensitivity analysis of CGPM entropy threshold on HCC-TACE-Seg.

CGPM entropy threshold	Prototype-eligible voxels (%) ↑	Dice (%) ↑	NSD (%) ↑	HD95 (mm) ↓	FP/case ↓	SACE ↓
τ*_h_* = 0.10	3.8	78.6	69.0	9.8	0.76	0.152
τ*_h_* = 0.15	5.1	78.8	69.2	9.7	0.75	0.150
τ*_h_* = 0.20	6.3	**79.0**	**69.5**	**9.6**	**0.74**	**0.149**
τ*_h_* = 0.25	7.8	78.9	69.3	9.6	0.76	0.151
τ*_h_* = 0.30	9.5	78.7	69.1	9.7	0.78	0.154
Adaptive Percentile Threshold	6.7	78.8	69.2	9.7	0.75	0.150

Bold values indicate the best performance among all compared methods or settings. ↑ and ↓ indicate that higher and lower values are better, respectively.

### 4.4 Visualization and mechanistic analysis

To provide a concise yet mechanistically informative visual assessment, we present three complementary visualization analyses on one representative case. Specifically, we examine slice-wise segmentation outputs on selected slices, the continuity of tumor area variation along one spatial dimension, and the corresponding entropy and prototype similarity maps. This design better matches the objectives of the proposed model than a large qualitative gallery, because it directly reflects boundary delineation, cross-slice consistency, and prototype-guided decision refinement.

**Figure 2:**
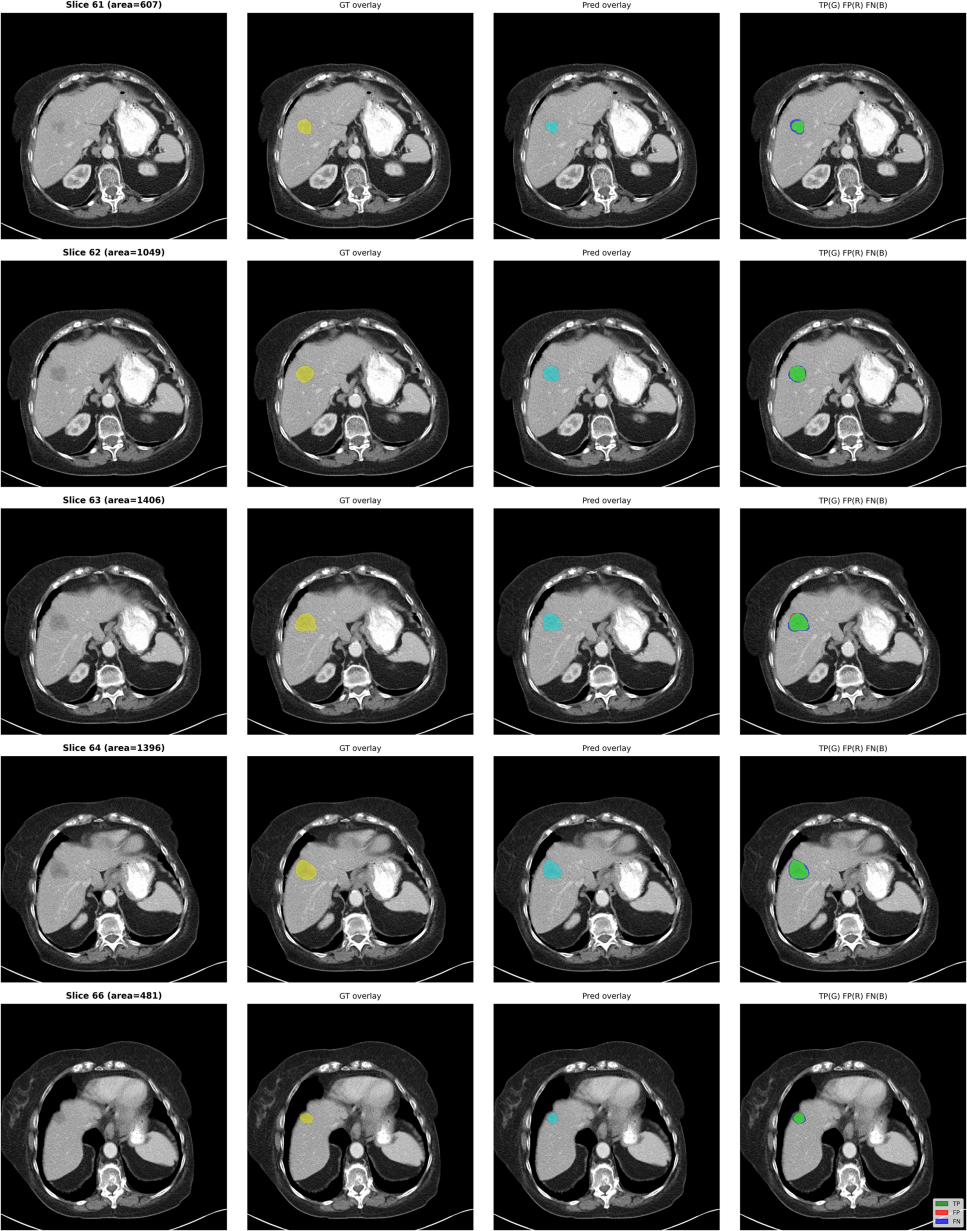
Slice-wise segmentation visualization on a representative case. Five slices, 61, 62, 63, 64, and 66, are selected from the same case, where the tumor area ranges from 481 to 1406 pixels. For each slice, the figure shows the portal-venous CT image, the ground truth, and the prediction of MemSAM-2.5D. The predicted masks remain well aligned with the reference annotation across consecutive slices, indicating stable delineation of lesion extent and boundary morphology under progressive lesion enlargement.

**Figure 2** presents the slice-wise segmentation results on five selected slices from one representative case. Across these slices, the tumor area ranges from 481 to 1406 pixels, providing a suitable example for evaluating model behavior under progressive lesion expansion and morphological variation. As the lesion enlarges and its contour becomes more irregular, the predicted masks remain well aligned with the ground truth and preserve the overall lesion extent with stable boundary delineation. These examples suggest that the proposed framework can maintain consistent segmentation quality across consecutive slices despite substantial changes in lesion size and shape.

**Figure 3:**
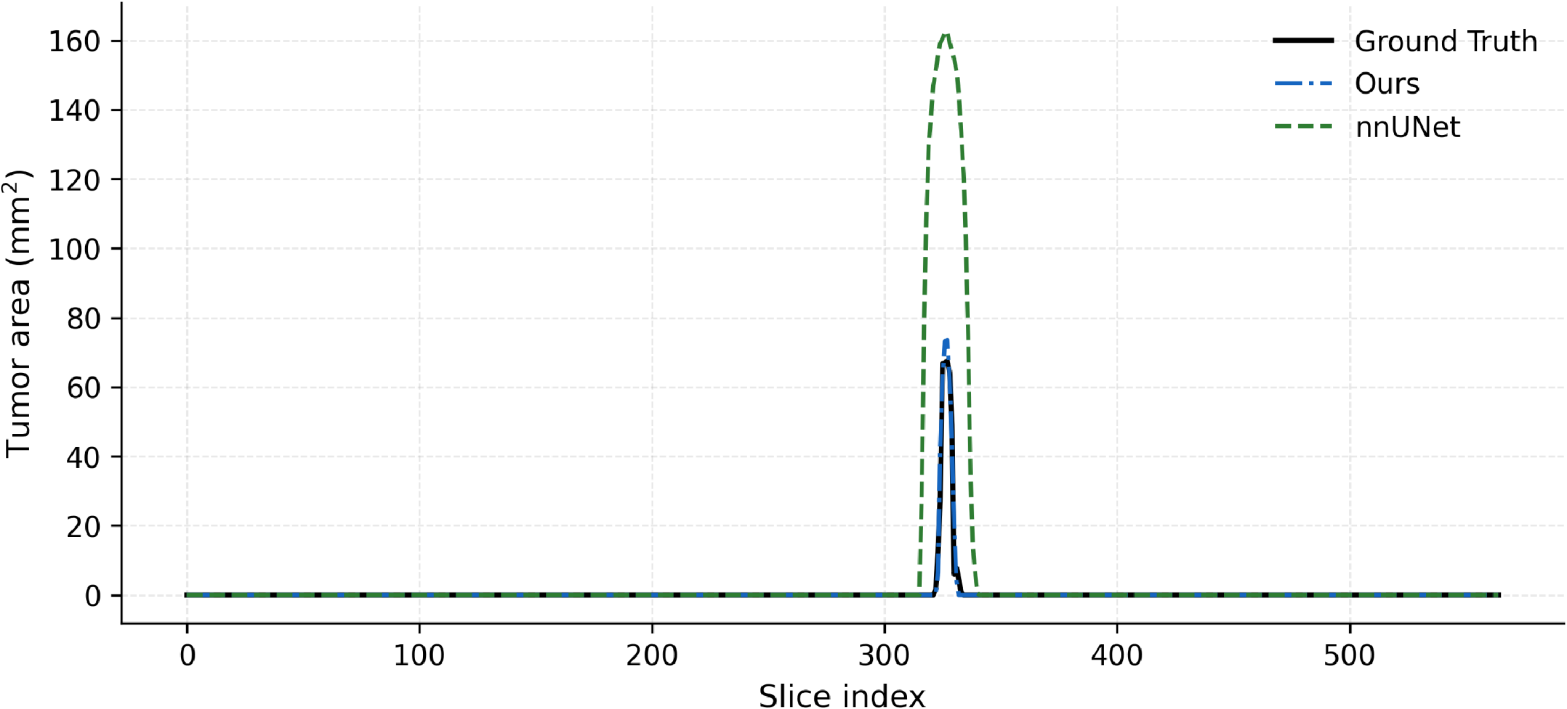
Tumor area variation along one spatial dimension for the same representative case. The x-axis denotes slice index and the y-axis denotes tumor area in pixels. Compared with nnU-Net, MemSAM-2.5D follows the ground-truth trend more closely and exhibits fewer abrupt oscillations, indicating better crossslice continuity and more stable volumetric behavior.

**Figure 3** further evaluates the same case from a volumetric continuity perspective. The proposed method follows the ground-truth tumor-area trajectory more closely than nnU-Net, particularly near lesion emergence, expansion, and regression. This behavior is consistent with the intended role of ZSF: by propagating a compact tumor-aware latent state across adjacent slices, the model suppresses abrupt area oscillations that commonly arise from independent slice-wise prediction. Therefore, **Figure 3** provides visual evidence that the proposed z-axis state propagation improves inter-slice consistency rather than merely increasing voxel-wise overlap.

**Figure 4:**
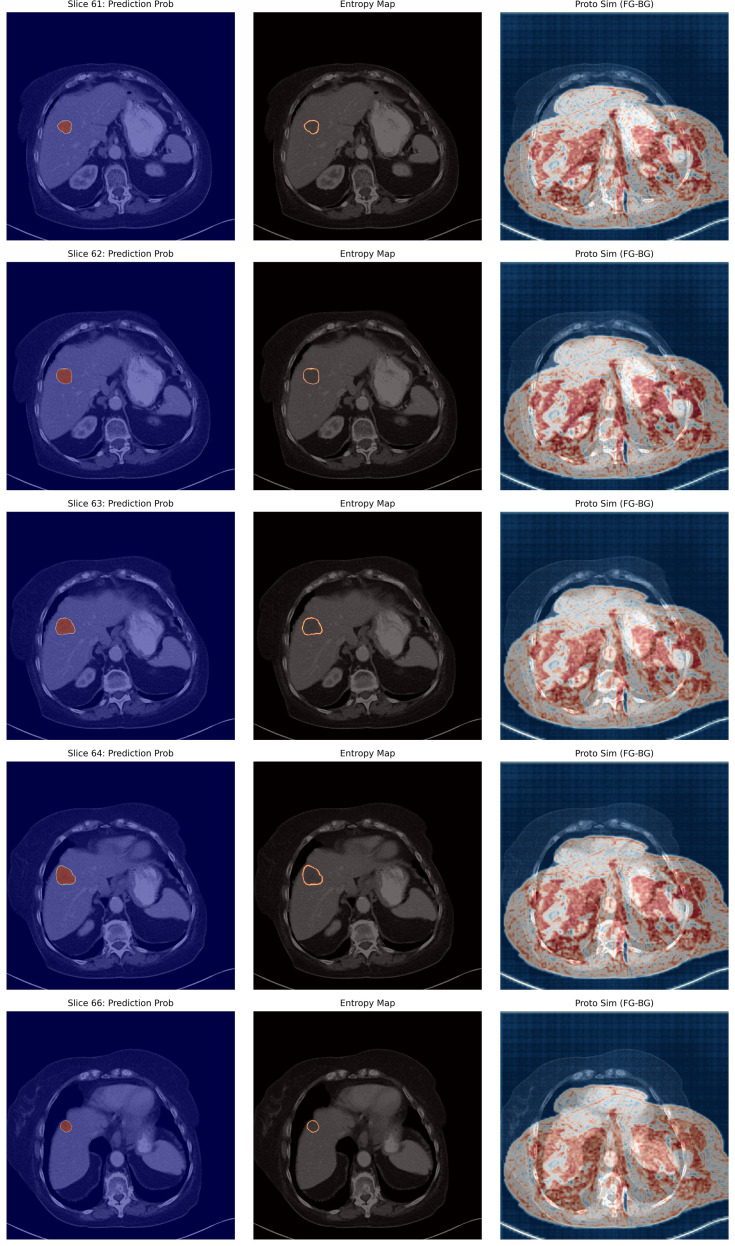
Entropy and prototype-response visualization for the same five slices shown in **Figure 2**. For each slice, the figure presents the CT image, the entropy map, the foreground prototype similarity map, and the background prototype similarity map. High-uncertainty responses are mainly concentrated around ambiguous lesion boundaries, while the foreground prototype similarity is concentrated in the tumor core and the background prototype similarity is stronger in surrounding non-tumor regions. This pattern suggests that the prototype memory provides discriminative guidance for resolving uncertain boundary regions rather than merely enlarging the foreground response.

To further understand the decision process of the model, **Figure 4** visualizes the entropy map together with the foreground and background prototype similarity maps for the same five slices shown in **Figure 2**. **Figure 4** provides mechanistic evidence for CGPM. High entropy is concentrated around lesion boundaries, whereas foreground prototype similarity is concentrated in the tumor core and background prototype similarity is stronger in surrounding non-tumor tissue. This spatial pattern indicates that CGPM mainly corrects uncertain boundary regions instead of indiscriminately enlarging the foreground mask. Combined with the Z-axis continuity pattern in **Figure 3**, these visualizations show that MemSAM-2.5D improves volumetric behavior through two coordinated mechanisms: ZSF stabilizes inter-slice tumor-state propagation, while CGPM suppresses boundary ambiguity using reliable class prototypes.

To further quantify the interaction among the proposed modules, we measured three mechanism-oriented indicators: tumor-state foreground/background contrast, adjacent-slice tumor-state correlation, and mean boundary entropy. These indicators correspond to the expected roles of HMA, ZSF, and CGPM, respectively. HMA is expected to improve tumor/background separability, ZSF is expected to improve state continuity across adjacent slices, and CGPM is expected to reduce uncertainty around ambiguous tumor boundaries.

As shown in **Table 12**, adding HMA substantially increased tumor-state foreground/background contrast, indicating that the hybrid local-global adapter improves discriminative intra-slice representation. Adding ZSF further increased adjacent-slice tumor-state correlation and markedly reduced SACE, supporting its role in stabilizing z-axis state propagation. Finally, the full model achieved the lowest mean boundary entropy, consistent with the uncertainty-guided correction behavior of CGPM. These results support the interpretation that the three modules are sequentially coupled: HMA improves the quality of the tumoraware state, ZSF stabilizes its propagation across slices, and CGPM resolves the remaining high-entropy boundary ambiguity.

**Table 12:** Quantitative mechanistic analysis of HMA, ZSF, and CGPM on HCC-TACE-Seg.

Variant	Tumor-state foreground/background contrast ↑	Adjacent-slice tumor-state correlation ↑	Mean boundary entropy ↓	SACE ↓
MedSAM-B baseline	0.18	0.67	0.46	0.214
+ HMA	0.27	0.75	0.42	0.203
+ HMA + ZSF	0.31	0.86	0.39	0.158
Full Model	**0.34**	**0.87**	**0.32**	**0.149**

Tumorstate foreground/background contrast measures the normalized separation between tumor-region and background-region state responses. Adjacent-slice tumor-state correlation measures the mean Pearson correlation of tumor-aware latent states between neighboring slices. Mean boundary entropy is computed over boundary-band voxels around the ground-truth tumor contour.

Bold values indicate the best performance among all compared methods or settings. ↑ and ↓ indicate that higher and lower values are better, respectively.

### 4.5 Deployment analysis and failure modes

To further characterize deployment behavior, we evaluated the reliability of the automatic liver-ROI localization stage, quantified robustness to controlled liver-ROI perturbations, and benchmarked inferencetime resource consumption. **Table 13** shows that the liver localizer was generally reliable, but failures were concentrated in peripheral lesions, especially small peripheral tumors in WAW-TACE. This directly identifies an important deployment failure mode: when the automatic liver ROI tightly follows the liver boundary, small peripheral lesions may be partially truncated, causing a measurable Dice drop. This motivates future lesion-aware ROI expansion or uncertainty-triggered prompt enlargement.

**Table 13:** Liver-localizer failure-mode analysis.

Dataset/subgroup	Cases	Tumor fully contained in automatic ROI (%) ↑	Partial truncation cases	Dice drop vs. ground-truth ROI
HCC-TACE-Seg, all cases	10	100.0	0/10	0.4
HCC-TACE-Seg, peripheral tumors ≤5 mm from ROI boundary	3	100.0	0/3	0.7
HCC-TACE-Seg, small peripheral tumors<10 mm	2	100.0	0/2	0.9
WAW-TACE, all cases	74	95.9	3/74	0.7
WAW-TACE, peripheral tumors ≤5 mm from ROI boundary	19	84.2	3/19	1.6
WAW-TACE, small peripheral tumors<10 mm	8	75.0	2/8	2.4

↑ indicates that higher values are better.

While **Table 13** evaluates naturally occurring liver-localizer failures, we further performed a controlled perturbation analysis to quantify how predefined localization errors affect tumor segmentation performance.

As shown in **Table 14**, MemSAM-2.5D was relatively stable under mild ROI perturbation, with only limited Dice degradation under a 5% box shift. However, tight cropping caused a larger performance drop than simple shifting, especially at the 10% perturbation level. This indicates that the main risk of liver-box localization error is not moderate contextual displacement, but partial exclusion of peripheral tumor regions.

**Table 14:** Sensitivity to controlled liver-ROI localization errors.

ROI perturbation	HCC-TACE-Seg dice (%) ↑	Dice drop	WAW-TACE dice (%) ↑	Dice drop
No perturbation	79.0	0.0	77.1	0.0
5% box shift	78.3	0.7	76.2	0.9
10% box shift	76.8	2.2	74.4	2.7
5% tight cropping	77.6	1.4	75.4	1.7
10% tight cropping	74.8	4.2	72.3	4.8

↑ indicates that higher values are better.

The degradation was slightly larger on WAW-TACE than on HCC-TACE-Seg, which is consistent with its more challenging low-tumor-burden distribution and external-domain setting.

As shown in **Table 15**, MemSAM-2.5D achieved substantially lower inference peak GPU memory than nnU-Net and U-Mamba, while maintaining comparable total inference time. This reflects the deployment trade-off of the proposed 2.5D design: it avoids dense 3D feature-volume storage by propagating a compact z-axis state, but its slice-wise sequential propagation prevents it from being substantially faster than optimized 3D sliding-window inference.

**Table 15:** Inference-time and peak-memory benchmark.

Method	Inference type	Mean processed ROI slices	Total time per liver-ROI volume (s) ↓	Effective latency per ROI slice (ms) ↓	Inference peak GPU memory (GB) ↓
nnU-Net	3D sliding-window inference	92	7.6	82.6	11.8
U-Mamba	3D CNN–SSM inference	92	8.4	91.3	13.6
MemSAM-2.5D	Sequential 2.5D inference with ZSF	92	8.1	88.0	5.2

Total inference time was measured over the cropped liver-ROI slice sequence rather than the full raw CT volume. Peak GPU memory refers to inference peak memory and is therefore distinct from the training peak memory reported in the adapter comparison.

↓ indicates that lower values are better.

## 5 Discussion

Accurate liver tumor segmentation in CT volumes remains challenging because lesions often exhibit substantial scale variation, weak or infiltrative boundaries, and strong continuity across adjacent slices. In this study, we proposed MemSAM-2.5D, a unified framework built upon MedSAM to address these three challenges through hybrid intra-slice representation learning, cross-slice state propagation, and uncertaintyguided prototype refinement. Across both the general liver tumor benchmark and the HCC-specific cohorts, the proposed method consistently achieved the best overall performance. Importantly, the gains were not limited to region-overlap metrics, but were also reflected in boundary-sensitive and lesion-level metrics, suggesting that the proposed design improves both geometric accuracy and the clinical plausibility of volumetric predictions.

Although HMA, ZSF, and CGPM are implemented as distinct modules, their functions are sequentially coupled. HMA first reshapes the MedSAM feature space by combining local texture-sensitive responses with global state-space context, thereby improving the foreground/background separability of the tumoraware representation. This enhanced feature space provides a cleaner input for ZSF, because the coarsemap-guided pooling is less likely to aggregate background-dominated features. ZSF then propagates the resulting compact tumor-aware state along the z-axis, reducing abrupt inter-slice prediction changes and producing a more coherent volumetric trajectory. CGPM operates after this propagation stage and focuses on the remaining high-entropy boundary regions, where it uses reliable tumor and background prototypes to correct uncertain logits. The expanded visualization and mechanistic analysis in Section 4.4 support this interpretation: **Figure 3** shows improved inter-slice area continuity, **Figure 4** shows prototype-guided correction around uncertain boundaries, and the quantitative mechanism analysis further shows increased tumor/background contrast, stronger adjacent-slice state correlation, and reduced boundary entropy.

A first important finding of this work is that direct transfer of a medical foundation model is beneficial, but insufficient for the complexity of HCC-focused CT segmentation. In our experiments, MedSAM-B already provided a reasonable baseline, indicating that large-scale medical pretraining offers useful general anatomical and semantic priors. However, its performance still lagged behind the proposed method on both MSD08 and the HCC-specific cohorts. This gap suggests that generic foundation-model priors alone cannot fully address the scale diversity of lesions, the sequential dependency of volumetric imaging, or the ambiguity of tumor margins. Therefore, task-oriented adaptation remains necessary when transferring promptable foundation models to clinically difficult oncologic imaging tasks.

Second, the results support the view that enhanced intra-slice representation is the foundation of robust HCC segmentation. The component-wise ablation study showed that adding HMA alone produced the largest single gain in Dice and also improved lesion-wise F1, indicating that better multi-scale feature representation directly benefits both tumor delineation and lesion detection. This observation is consistent with the heterogeneous appearance of HCC lesions in portal-venous CT, where small lesions require preservation of fine local texture, whereas larger or irregular tumors require broader contextual modeling. The size-stratified analysis further supports this interpretation, as MemSAM-2.5D improved both smalllesion recall and large-lesion Dice compared with the MedSAM-B + CNN Adapter baseline. These findings suggest that the local convolutional branch and the global state-space branch are complementary rather than redundant.

Third, our findings indicate that explicit modeling of volumetric continuity is important even in an efficient 2.5D setting. Slice-wise models often produce fragmented predictions because adjacent slices are processed largely independently. In contrast, the proposed ZSF module propagates a compact tumoraware latent state along the z-axis and uses it to modulate the current-slice features before decoding. The ablation study showed that adding ZSF resulted in the most pronounced reduction in SACE, suggesting that its principal effect is to stabilize inter-slice behavior rather than merely increase regional overlap. This interpretation is further supported by the visualization analysis, in which the predicted tumor-area trajectory followed the ground-truth trend more closely and exhibited fewer abrupt oscillations than the baseline. From a clinical perspective, such continuity is highly relevant because implausible slice-to-slice fluctuations can reduce radiologist trust and may compromise downstream volumetric assessment.

Fourth, the proposed uncertainty-guided prototype refinement appears particularly effective for suppressing false positives in ambiguous regions. HCC lesions frequently occur near vessels, heterogeneous liver parenchyma, or infiltrative margins, where deterministic predictions are often unstable. In our framework, CGPM selectively writes only high-confidence and low-uncertainty features into the prototype memory and then uses these reliable prototypes to refine uncertain regions during inference. The component-wise ablation showed that CGPM substantially reduced FP/case, while the entropy and prototype-similarity visualizations suggested that the module mainly acts around uncertain lesion boundaries rather than indiscriminately enlarging the foreground region. This behavior is clinically meaningful because falsepositive foci and boundary over-segmentation are common failure modes in liver tumor segmentation and can mislead the assessment of tumor burden or treatment response.

Another notable result is the robustness of MemSAM-2.5D on the external WAW-TACE cohort. Compared with the internal HCC-TACE-Seg cohort, WAW-TACE contains a much lower tumor pixel ratio and presents a more challenging test distribution. Although all methods showed some performance decline on the external cohort, the proposed method maintained a relatively stable advantage across Dice, NSD, HD95, and lesion-wise F1. This suggests that the gains are not merely due to adaptation to a single institutional distribution, but instead arise from more transferable feature modeling and refinement mechanisms. Such robustness is particularly important for HCC applications, because real-world deployment inevitably involves cross-center differences in acquisition protocol, lesion appearance, and foreground sparsity.

The present study also has several limitations. First, although the method was evaluated on one public benchmark and two HCC-specific cohorts, the overall sample size remains moderate, especially for external validation. The current external validation relies on one independent HCC cohort, WAW-TACE. Although WAW-TACE provides a meaningful cross-center evaluation and contains a challenging low-tumor-burden distribution, a third external HCC cohort would further strengthen the evidence for multicenter robustness. We were unable to include an additional annotated external HCC cohort in the current revision because no further portal-venous phase CT cohort with compatible voxel-level tumor annotations was available under the current data-use approval. Future work will prioritize validation on additional multicenter HCC cohorts with different scanners, acquisition protocols, and treatment backgrounds. Second, all HCC-specific experiments were conducted using the portal-venous phase only. This design was adopted because the portal-venous phase was consistently available across the evaluated HCC cohorts and has been commonly used in recent liver tumor segmentation benchmarks. Nevertheless, multiphase CT is clinically important for HCC characterization because arterial enhancement and delayed washout may provide complementary lesion cues. Technically, MemSAM-2.5D can be extended to multiphase CT by adding a phase-wise state propagation module parallel to the current z-axis ZSF. In such a design, each phase could first be encoded by the HMA-enhanced MedSAM encoder, and a temporal or phase-wise ZSF module could then model arterial-to-portal-venous-to-delayed phase dependencies. This would allow the model to incorporate enhancement dynamics while preserving the memory-efficient 2.5D formulation. We therefore regard multiphase extension as an important direction for future work. Third, the current framework depends on an automatically generated liver ROI box. Although the localizer was reliable in most cases and the controlled perturbation analysis showed robustness to mild box shifts, peripheral lesions, especially small peripheral lesions in the external cohort, can still be partially truncated when the ROI tightly follows the liver boundary. Moreover, controlled shift and cropping perturbations approximate common liver-box localization errors but cannot cover all possible localizer failures, such as irregular liver-mask boundaries or z-axis truncation near the hepatic dome and inferior liver edge. Fourth, the sequential 2.5D design is memory-efficient but not necessarily faster than optimized 3D inference, because Z-axis state propagation is performed slice by slice. Fifth, CGPM may suffer from confirmation bias when the initial decoder prediction is confidently incorrect. In the current implementation, this risk is mitigated by requiring both high prediction probability and low entropy before memory writing. However, this gating strategy cannot fully eliminate systematic high-confidence errors. Future work should investigate confidence-decay or memory-reset mechanisms, in which the prototype bank is partially down-weighted or cleared when abrupt foreground-area changes, high global entropy, or inconsistent adjacent-slice states indicate possible tracking failure.

Future work may proceed in several directions. One promising direction is to extend the current framework to multiphase CT or multimodal imaging so that complementary enhancement patterns can be incorporated into the segmentation process. Another is to investigate more adaptive prompting strategies, including lesion-aware prompts or joint liver–tumor structural priors, to further exploit the promptable nature of SAMbased architectures. It would also be valuable to validate the method prospectively in larger multicenter cohorts and to examine whether improved segmentation translates into better performance in downstream tasks such as treatment response monitoring, prognostic modeling, and surgical or interventional planning.

Overall, the proposed framework demonstrates that a medical foundation model can be more effectively adapted to volumetric liver tumor segmentation when multi-scale lesion variability, inter-slice continuity, and boundary ambiguity are addressed in a coordinated manner. The consistent gains observed across public, internal, and external datasets indicate that integrating hybrid representation learning, sequential state propagation, and uncertainty-aware prototype correction is a promising direction for clinically reliable HCC segmentation.

## 6 Conclusions

In this study, we proposed MemSAM-2.5D, a unified 2.5D framework for liver tumor segmentation in CT volumes that builds upon the MedSAM foundation model and introduces three complementary components: the Hybrid Mamba-Adapter for multi-scale intra-slice representation, the Z-axis State Flow module for interslice continuity modeling, and the Confidence-Gated Prototype Memory for ambiguity-aware boundary refinement. Extensive experiments on MSD08, HCC-TACE-Seg, and WAW-TACE demonstrated that the proposed method consistently outperformed representative CNN-based, Transformer-based, Mamba-based, and MedSAM-based baselines. These improvements were reflected not only in overlap-based metrics, but also in boundary-sensitive, lesion-level, and continuity-related measures. Taken together, the results suggest that MemSAM-2.5D provides an effective and transferable solution for clinically relevant HCC segmentation and offers a practical direction for adapting promptable medical foundation models to challenging volumetric oncologic imaging tasks.

## Data Availability

The original contributions presented in the study are included in the article/supplementary material. Further inquiries can be directed to the corresponding authors. The datasets analyzed in this study are publicly available from their original repositories. The MSD08 dataset from the Medical Segmentation Decathlon can be accessed through the Medical Segmentation Decathlon/AWS Open Data repository at https://registry.opendata.aws/msd/ and https://medicaldecathlon.com/. The LiTS 2017 dataset can be accessed through the Liver Tumor Segmentation Challenge repository at https://academictorrents.com/details/27772adef6f563a1ecc0ae19a528b956e6c803ce.
